# Comparative analysis of the performance of the large language models DeepSeek-V3, DeepSeek-R1, open AI-O3 mini and open AI-O3 mini high in urology

**DOI:** 10.1007/s00345-025-05757-4

**Published:** 2025-07-07

**Authors:** Zijun Yan, Ke-qin Fan, Qi Zhang, Xinyan Wu, Yuquan Chen, Xinyu Wu, Ting Yu, Ning Su, Yan Zou, Hao Chi, Liangjing Xia, Qiang Cao

**Affiliations:** 1 Department of Pharmacy, Panzhihua Central Hospital, Panzhihua, 617067 Sichuan China; 2 College of Chinese Medicine, Hong Kong Baptist University, HKG, Kowloon Tong, Hong Kong; 3 College of Veterinary Medicine, Sichuan Agricultural University, Chengdu, 610000 China; 4 School of Public Health and Preventive Medicine, Faculty of Medicine, Nursing & Health Sciences, Monash University, Level 1, 553 St Kilda Road, Melbourne, VIC 3004 Australia; 5 School of Pharmaceutical Sciences, Yunnan Key Laboratory of Pharmacology for Natural Products, Kunming Medical University, Kunming, 650500 Yunnan China; 6 School of Clinical Medicine, Southwest Medical University, Luzhou, 402103 China; 7 Department of Earth Sciences, Kunming University of Science and Technology, Kunming, 650093 China

**Keywords:** Urology, Large language models, Clinical guidelines, Performance evaluation, Self‑correction capacity

## Abstract

**Objectives:**

We sought to compare how DeepSeek‑V3, DeepSeek‑R1, OpenAI o3‑mini, and OpenAI o3‑mini high handle urological questions, especially in areas such as benign prostatic enlargement, urinary stones, infections, and guideline updates. The intent was to identify how these text‑creation platforms might aid clinical practice without overlooking potential gaps in accuracy.

**Methods:**

A set of 34 routinely asked questions plus 25 queries based on newly revised guidelines was assembled. Six board‑certified urologists independently scored each system’s replies using a five‑point scale. Questions scoring below a set threshold were reintroduced to the same system, accompanied by critiques, to gauge self‑correction. Statistical analyses focused on total scores, percentage of excellent ratings, and improvements after iterative prompting.

**Results:**

Across all 59 queries (34 general plus 25 guideline-based), OpenAI o3-mini high recorded the highest median total score (22 [20–24]), significantly outperforming DeepSeek-R1, DeepSeek-V3 and OpenAI o3-mini (all pair-wise *p* < 0.01). DeepSeek-R1’s accuracy approached that of o3-mini high in patient-counseling items, where their excellent-answer rates were 49% and 57%, respectively. DeepSeek‑V3 achieved solid baseline correctness but made fewer successful corrections on subsequent attempts. Although OpenAI o3‑mini initially produced more concise responses, it showed a surprisingly strong capacity to revise earlier errors.

**Conclusion:**

OpenAI o3‑mini high, followed by DeepSeek‑R1, provided the most reliable answers for modern urological concerns, whereas DeepSeek‑V3 exhibited limited adaptability during re‑evaluation. Despite often briefer replies, OpenAI o3‑mini outdid DeepSeek‑V3 in self‑correction. These findings indicate that, when reviewed by a clinician, o3-mini high can serve as a rapid second-opinion tool for outpatient counselling and protocol updates, whereas DeepSeek-R1 may provide a cost-effective alternative in resource-limited settings.

**Supplementary Information:**

The online version contains supplementary material available at 10.1007/s00345-025-05757-4.

## Introduction

Urology, as a distinct branch of medical practice, covers a surprisingly wide domain of concerns related to the urinary tract and the male reproductive system. In day-to-day practice, a urologist deals not only with kidneys, ureters, and bladders but also with prostates, testicles, and assorted pelvic organs [1–7]. With developments such as endoscopic fiber-optic technology and advanced imaging protocols, the once-rudimentary landscape of kidney stone removal, bladder tumor resection, or prostatic surgery has transformed into a high-tech environment. Yet, these technological leaps bring fresh complexities. For instance, the introduction of robotic-assisted laparoscopic prostatectomy improved surgical precision but added a steeper learning curve, not to mention cost considerations for institutions [8–14]. Training, mentorship, and standardization of care are vital for ensuring good clinical results in procedures like transurethral prostate resection or radical cystectomy. At the same time, urology remains intimately concerned with patients’ quality of life, addressing sexual function, incontinence, chronic pelvic pain, and psychosocial factors that are not always easy to quantify.

In recent years, there has been a global wave of interest in large language model that generate written content with remarkable fluency. Urologists, who routinely sift through massive amounts of clinical data and evolving scientific literature, have taken notice of these emerging text-processing platforms [15–21]. To date, however, no head-to-head study has simultaneously benchmarked proprietary and open-source LLMs against expert opinion on both everyday and guideline-driven urological decision points, leaving a clear gap in evidence for their comparative clinical utility. Among them, DeepSeek-V3 and DeepSeek-R1 have garnered considerable attention within academic communities for their ability to produce elaborate, context-aware narratives, sometimes matching or exceeding what a human peer would generate when asked about key issues like prostate cancer screening or surgical guidelines for kidney stones. DeepSeek-V3, known for its enormous parameter count, has stirred curiosity because of its emphasis on mixture-of-experts structure, allegedly allowing for more nuanced, specialized replies in logic-heavy or coding-related contexts—though some experts question whether it tends to lack deeper reasoning in certain nuanced clinical scenarios [22]. DeepSeek-R1, which shares a similar foundation, introduces a reinforcement-based approach intended to refine the clarity and correctness of its results, potentially making it more transparent in the way it formulates answers to tricky queries such as deciding when to offer radical cystectomy [23]. Alongside these DeepSeek systems, the Open AI-O3 mini versions have also gained a foothold worldwide, especially among clinicians seeking a more nimble text generator with robust question-answering capabilities in science, math, and stepwise reasoning. DeepSeek models rely on a Mixture-of-Experts architecture that routes each question to specialised sub-networks, trading raw speed for greater topical depth, whereas the OpenAI o-series employs a dense-transformer backbone tuned heavily on reasoning and safety alignment to deliver concise, broadly reliable answers. Readers unfamiliar with these platforms can freely experiment with demo endpoints provided at https://platform.deepseek.com and https://platform.openai.com. The O3 mini high boasts a higher reasoning level, offering, in principle, more precise solutions for complicated quandaries one might face in advanced oncologic guidelines or intricate reconstructive surgery decisions [24, 25]. According to international medical discourse, these text-producing tools hold the promise of accelerating data assimilation, summarizing newly released guidelines, and educating trainees who lack the time to comb through every detail in voluminous clinical trial reports [26–31]. However, consistent reliability has not been proven in every domain, and some practitioners worry that partial inaccuracies—especially around antibiotic stewardship or novel hormone therapy intervals—could inadvertently influence clinical judgment. For instance, early deployments of chat-based triage systems recommended fluoroquinolones for uncomplicated cystitis despite 2019 FDA safety warnings, and a dermatology-focused LLM mislabelled histologically proven early melanomas as benign naevi, underscoring the tangible risks of unquestioned AI output [32–34].Artificial-intelligence deployment in health care therefore raises a distinct set of ethical obligations that reach beyond traditional notions of clinical accuracy. First, training-data bias can silently propagate health-care inequities if a model is fine-tuned on datasets that under-represent certain age groups, ethnicities, or resource-limited settings. Second, privacy and data-protection statutes such as the European Union’s GDPR and China’s Personal Information Protection Law mandate explicit safeguards when patient notes or imaging data are uploaded for model refinement. Third, explainability is no longer an academic luxury: without transparent rationales clinicians cannot satisfy the duty of informed consent, nor can they contest unsafe recommendations in medico-legal disputes. Finally, most regulatory frameworks—including the U.S. FDA’s proposed AI/ML-Enabled Device guidance—now emphasise “human-in-the-loop” accountability, requiring that automated suggestions be auditable and overruled by a licensed practitioner. Any evaluation of large language models for urology must therefore consider not only numerical performance but also these intertwined issues of bias mitigation, privacy preservation, traceability, and professional responsibility [35–41]. The worldwide situation thus stands at a crossroads: many teaching hospitals and research centers are experimenting with DeepSeek or Open AI-O3 platforms for academic writing support, patient education materials, and quick reference queries, but the debate regarding authenticity, interpretative errors, and liability for misguided care remains lively.

This article attempts to compare these text‑creation systems—DeepSeek‑V3, DeepSeek‑R1, OpenAI o3‑mini, and OpenAI o3‑mini high—through a structured set of questions drawn from everyday urological practice and from newly revised guidelines. By enlisting expert evaluation of each system’s responses and exploring how well they self‑correct after pointed feedback, we hope to illuminate both the benefits and drawbacks these systems might present in clinical environments.

## Methods

### Study design

A set of frequently encountered clinical questions pertaining to urological conditions was assembled by reviewing reputable online medical resources and standard clinical guidelines. The assembled questions encompassed common urological complaints, diagnostic approaches, therapeutic strategies, preventive measures, and emerging trends in urology. To capture current practice scenarios, the question set was refined by experienced urologists who prioritized queries deemed most representative of everyday clinical decision-making and patient education in urology. In total, 34 questions were identified as reflective of routine urological practice, addressing conditions such as benign prostatic hyperplasia, urinary tract infections, nephrolithiasis, oncological concerns (e.g., prostate cancer), and postoperative management. These questions were organized into thematic groups to facilitate focused assessment: pathology and mechanisms, diagnosis, treatment, prevention, and patient counseling, as shown in Appendix Table 1. An additional set of 25 questions was derived from recent updates to urological practice guidelines—particularly those released in the past 18 months by major associations—to evaluate how accurately each model handles the latest evidence-based recommendations [42–50]. Each question was presented individually to the four models—DeepSeek-V3, DeepSeek-R1, OpenAI o3-mini, and OpenAI o3-mini high—to solicit responses under identical conditions, as shown in Appendix Table 2.

**Table 1:** Length of responses for 34 general urology questions

Model	Word count, M(P25–P75)	Character count, M(P25–P75)
DeepSeekV3	292.00 (271.00–329.00)	2108.00 (1822.00–2414.00)
DeepSeekR1	288.00 (252.50–320.00)	2004.50 (1746.75–2136.00)
OpenAI o3mini	252.50 (218.75–278.50)*	1598.00 (1443.75–1708.25)*
OpenAI o3mini high	269.00 (221.00–293.00)^	1786.00 (1625.50–1985.50)^

*P < 0.05 vs. DeepSeek‑V3, ^ *P* < 0.05 vs. DeepSeek‑R1

**Table 2:** Length of responses for 25 guideline‑based questions

Model	Word count, M(P25–P75)	Character count, M(P25–P75)
DeepSeekV3	287.50 (265.00–316.00)	2035.50 (1774.00–2254.00)
DeepSeekR1	302.00 (285.00–335.00)	2136.00 (1986.50–2342.00)
OpenAI o3mini	250.00 (210.00–275.50)*	1527.00 (1356.00–1693.50)*
OpenAI o3mini high	274.50 (232.00–291.50)	1672.00 (1534.75–1853.00)

* *P* < 0.05 vs. DeepSeek‑R1

### Expert evaluation and scoring

Six board-certified urology experts (median practice 14 years, range 11–22), recruited through the national society’s peer-review roster to minimise institutional bias, independently appraised each model’s responses. Six board-certified urology experts—actively practising clinicians with no financial relationship to the companies developing the evaluated models and excluded if they had co-authored AI-related commercial work—independently appraised each model’s responses.The evaluation was blinded such that the experts were unaware of which model produced the text. To ensure a clear differentiation from previously published rating methods, a customized five-point scoring scale was adopted:

1. 1 point—factually incorrect or highly misleading content.
2. Points—partially accurate but containing serious omissions or errors.
3. Points—generally accurate but lacking important details or clinical nuance.
4. Points—accurate, appropriately detailed, and clinically relevant.
5. Points—comprehensive, precise, and demonstrating superior clinical applicability.

Each expert awarded a separate score (ranging from 1 to 5) for every question response from the four models, yielding a total score out of 30 (5 points × 6 experts) per query. When two reviewers’ ratings for a given response differed by more than two points, the full panel convened a brief blinded tele-conference and reached consensus by majority vote, ensuring a uniform final score without altering individual rater statistics used for Fleiss’ κ. For interpretive consistency, overall performance was grouped into three categories: scores under 10 were labeled “Inadequate,” scores of 10 to 20 were “Acceptable,” and scores above 20 were “Excellent.”

### Prompting for self-correction

To investigate the capacity of each model to refine answers through iterative feedback, responses scoring below 10 (“Inadequate”) were presented again to the same model alongside a brief critique indicating the primary weaknesses or inaccuracies. The prompt encouraged the model to “review and correct any errors, then provide the most up-to-date, accurate response possible.” This new response was gathered in plain text form and re-evaluated by the same six experts in a separate round, conducted two weeks later. Experts were intentionally blinded to whether the response under review was an original or revised answer. The difference in scores before and after self-correction was then analyzed to quantify the model’s capacity for meaningful improvement.

### Statistical analysis

All raw scores from the six experts were collected and tested for interrater reliability. Fleiss’ kappa was used to gauge the level of agreement among the six evaluators regarding each model’s responses. Mean ± SD (or median [IQR]) were computed for each item; cross-model differences were tested with Kruskal–Wallis analysis followed by Bonferroni-adjusted Dunn pairwise tests, while before-versus-after self-correction scores were analysed with Wilcoxon signed-rank tests. Post hoc analyses, including pairwise comparisons, were carried out when overall comparisons suggested significant differences. Changes in scoring before and after self-correction were examined using paired tests, with statistical significance set at *p* < 0.05.

## Results

### Lengths of model responses

Table 1 compares median word- and character-counts for the 34 routine prompts. DeepSeek-V3 and DeepSeek-R1 produced the longest replies (≈ 290 words). o3-mini high was ~ 8% shorter and o3-mini ~ 14% shorter (both *p* < 0.05 vs. DeepSeek-V3). Character-count differences were concordant.

For the 25 guideline-based prompts (Table 2) DeepSeek-R1 wrote the longest answers (302 words [285–335]), DeepSeek-V3 was second (288 words [265–316]) and o3-mini the briefest (250 words [210–276], *p* < 0.05 vs. DeepSeek-R1). A post-hoc Spearman analysis revealed only a weak, non-significant association between response length and accuracy (ρ = 0.18, *p* = 0.12), suggesting that verbosity alone did not determine correctness.

### Accuracy on the 34 general-practice questions

Median total scores (TS) by thematic domain are presented in Table 3. o3-mini high led overall (22.0 [20.0–24.0]), significantly outperforming every comparator (*p* < 0.05). DeepSeek-R1 placed second and essentially tied o3-mini high for patient-counselling items (23.0 vs. 24.0, *p* = 0.062). o3-mini recorded the lowest general-question median (17.5), largely because its terse phrasing omitted qualifying details.

**Table 3:** Total scores (TS) for 34 general questions by thematic category

Category	DeepSeekV3, M(P25–P75)	DeepSeekR1, M(P25–P75)	OpenAI o3mini, M(P25–P75)	OpenAI o3mini high, M(P25–P75)	*P* value
Pathology and mechanisms	19.00 (17.00–21.00)	22.50 (20.00–23.00)	16.00 (14.00–18.50)	23.50 (21.00–25.00)*	0.006
Diagnosis	19.00 (16.50–20.50)	21.00 (19.00–22.50)	17.00 (14.75–19.00)	22.00 (20.00–24.00)	0.012
Treatment	20.00 (18.00–21.00)	21.50 (19.00–23.00)	18.50 (15.00–20.00)	24.00 (22.00–25.00)*	0.004
Prevention	19.50 (17.00–21.00)	20.00 (18.00–22.00)	17.50 (15.00–20.00)	22.00 (20.00–24.00)	0.028
Patient counseling	20.00 (18.00–22.00)	23.00 (20.75–24.00)	19.50 (17.00–21.50)	24.00 (22.00–25.00)	0.06
All general questions	19.50 (17.00–22.00)	20.50 (18.75–23.00)	17.50 (15.00–20.00)	22.00 (20.00–24.00)*	0.003

*P < 0.05 vs. all other models

### Accuracy on the 25 guideline-based questions

Table 4 details the guideline cohort. o3-mini high achieved the highest median TS—23.0 (21.0–25.0), range 17–30—and delivered an “Excellent” answer (> 20/30) for 60% of prompts. DeepSeek-R1 ranked second with a median 20.0 (19.0–23.0), range 15–29, performing particularly well on active-surveillance and imaging-interval questions but missing fine points on antibiotic prophylaxis. DeepSeek-V3 showed similar central tendency (19.5 [17.0–21.0]) yet was less consistent, occasionally providing conflicting dosing schedules. o3-mini trailed (16.5 [14.0–18.5]) because its brevity sacrificed guideline granularity; nevertheless, the model was never dangerously wrong, and 29% of its answers still reached the “Excellent” threshold.

**Table 4:** Total scores (TS) for 25 guideline-based questions

Model	Median (P25–P75)	Range
DeepSeekV3	19.50 (17.00–21.00)	13–26
DeepSeekR1	20.00 (19.00–23.00)	15–29
OpenAI o3mini	16.50 (14.00–18.50)	10–23
OpenAI o3mini high	23.00 (21.00–25.00)*	17–30

*P < 0.05 vs. all other models

### Global accuracy distribution

**Fig. 1:**
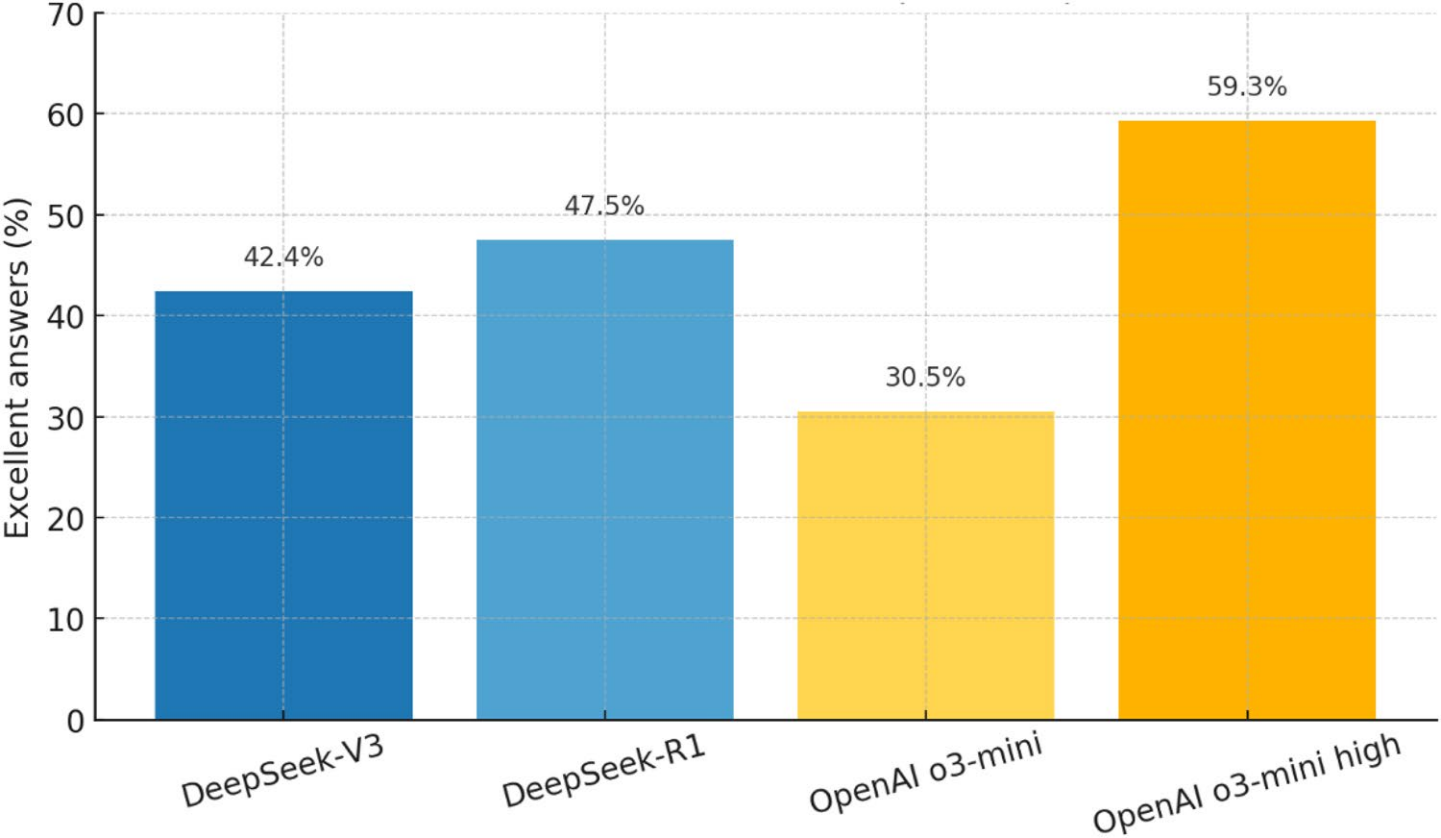
Proportion of “Excellent” (> 20/30) answers across 59 questions

Aggregating all 59 prompts, Table 5 shows that o3-mini high produced “Excellent” answers in 59% of cases and fell below “Acceptable” (< 10/30) only six times. Figure 1 visually confirms this gradient, highlighting the markedly higher share of ‘Excellent’ responses achieved by o3-mini high compared with the other three models. DeepSeek-R1 achieved 48% excellent, DeepSeek-V3 42%, and o3-mini 31%.

**Table 5:** Accuracy rating distribution for all 59 questions (34 general + 25 guideline-based)

Model	Poor < 10, *n* (%)	Acceptable 10–20, *n* (%)	Excellent > 20, *n* (%)
DeepSeek-V3	19 (32.2%)	15 (25.4%)	25 (42.4%)
DeepSeek-R1	11 (18.6%)	20 (33.9%)	28 (47.5%)
OpenAI o3-mini	23 (39.0%)	18 (30.5%)	18 (30.5%)
OpenAI o3-mini high	6 (10.2%)	18 (30.5%)	35 (59.3%)

### Self-correction ability

Among responses initially rated “Inadequate,” re-prompting yielded the consolidated outcomes in Table 6. Median scores for o3-mini high nearly doubled for both general (*p* = 0.014) and guideline (*p* = 0.013) prompts. Significant improvements were also seen for o3-mini and DeepSeek-R1, whereas DeepSeek-V3’s gains were modest and non-significant. Average point increases across all inadequate answers were: o3-mini high + 6.2, o3-mini + 5.2, DeepSeek-R1 + 4.7, DeepSeek-V3 + 2.1.

**Table 6:** Self-correction outcomes for all “inadequate” responses (< 10 points)

Model	General questions initial TS M (P25–P75) → Post TS	*p*-Value	Guideline questions initial TS M (P25–P75) → Post TS	*p*-Value
DeepSeek-V3	7.00 (5.75–8.25) → 9.00 (7.50–10.00)	0.096	6.00 (5.00–7.00) → 8.00 (6.50–9.00)	0.072
DeepSeek-R1	7.00 (6.00–8.00) → 12.50 (10.00–14.00)	0.022	8.00 (7.00–9.00) → 12.00 (11.00–13.00)	0.028
OpenAI o3-mini	6.50 (5.00–9.00) → 12.00 (10.00–13.00)	0.019	5.00 (3.00–7.00) → 11.00 (9.00–13.00)	0.015
OpenAI o3-mini high	8.00 (7.00–9.00) → 14.00 (12.00–15.00)	0.014	8.00 (7.00–8.00) → 15.00 (13.50–16.00)	0.013

### Inter-rater reliability

Expert agreement was moderate for general prompts (κ = 0.54, 95% CI 0.47–0.60) and strong for guideline prompts (κ = 0.68, 95% CI 0.61–0.75). Most disagreements occurred at the 19–21-point border between “Acceptable” and “Excellent.”

### Overall ranking

Considering first-pass accuracy (Tables 3 and 4) and feedback responsiveness (Table 6), the final hierarchy was: o3-mini high > DeepSeek-R1 > o3-mini > DeepSeek-V3. Even the lowest performer (o3-mini) maintained a median 17.5/30, so none of the four systems generated uniformly unsafe advice.

## Discussion

Traditionally, the assessment of automated text-generation tools in urology has centered on their capacity to provide accurate, clinically relevant, and trustworthy information. Because all six reviewers were academically affiliated, their preferences for formal medical prose may have favoured certain stylistic features; future work should include community-based urologists to mitigate this potential rating bias. In this comparative evaluation of DeepSeek-V3, DeepSeek-R1, OpenAI o3-mini, and OpenAI o3-mini high, the observed performance differences offer crucial insights into how these models might be harnessed in daily clinical scenarios. Clarity and precision in handling common urological conditions—such as benign prostatic hyperplasia, urinary tract infections, nephrolithiasis, and prostate cancer—are paramount. Notably, OpenAI o3-mini high displayed a particularly impressive level of thoroughness and depth in responses, achieving higher overall scores in various thematic categories. Such robust performance suggests potential benefits for complex decision-making, for example, in evaluating evolving treatments for metastatic prostate cancer or selecting best-practice methods for antibiotic prophylaxis. DeepSeek-R1 demonstrated commendable accuracy, sometimes approaching the proficiency of OpenAI o3-mini high, especially in domains like patient counseling. Its capacity to illuminate finer points in postoperative care and emerging interventions illustrates an aptitude for guiding nuanced discussion with patients. DeepSeek-V3, while solid in foundational knowledge, occasionally faltered when confronted with the need for detailed clarification of contemporary guidelines, and its self-correction feature appeared more limited. Conversely, OpenAI o3-mini stood out through concise communication that may be valuable in streamlining patient education, though it sometimes lacked the comprehensive breadth desired by specialists. These findings underscore the need for critical evaluation of generative tools that purport to enhance clinical practice.Comparable cross-disciplinary evaluations echo our results; in cardiology and dermatology benchmarks, higher-parameter OpenAI variants likewise surpassed Open-source MoE models in guideline fidelity, whereas smaller but well-aligned systems still improved clinician efficiency. Such concordance suggests that our urology-focused observations reflect a broader pattern rather than a specialty-specific anomaly. Automated answers alone cannot replace expert oversight, especially given the heterogeneity of patient presentations in real-world settings. Beyond accuracy, the ethical landscape includes safeguarding patient confidentiality, clarifying liability for AI-derived advice, and ensuring transparency when generative text is incorporated into clinical notes. All four models occasionally produced confident but spurious statements—an instance of hallucination that, if unrecognised, could propagate unsafe recommendations such as outdated antibiotic regimens; mandatory human verification therefore remains indispensable. The subtle differences in diagnostic pathways and recommended treatments require the measured discernment of a trained practitioner, informed by guidelines that evolve rapidly. The higher performance of certain systems in advanced oncologic settings can be a promising supplement in multidisciplinary teamwork, ensuring that key updates—such as new hormone therapy dosing intervals or altered imaging schedules—are not overlooked. However, even the models that excelled in accuracy and detail may exhibit inconsistencies when dealing with layered questions involving psychosocial factors. Consistent validation by urology professionals remains essential to prevent inaccuracies that might compromise patient outcomes. Ultimately, these observations highlight how technological innovations in language modeling, although promising, should be integrated thoughtfully, with vigilant clinical governance safeguarding the quality of care at every step.

DeepSeek-V3 and DeepSeek-R1 both utilize a Mixture of Experts (MoE) configuration, boasting large parameter counts that facilitate complex text generation. Notably, DeepSeek-R1’s emphasis on iterative reinforcement learning and group relative policy optimization contributes to an enhanced reasoning capacity and more transparent solution derivation in many tasks. This improved reasoning is evident when interpreting advanced urological guidelines, although it can sometimes lead to verbose explanations requiring further pruning for clarity. By contrast, the OpenAI o3-mini series, including the higher-intensity variant, optimizes for science, technology, engineering, and mathematics reasoning while maintaining cost-efficiency and speed—a design choice that manifests in concise but high-quality responses. The self-correction capacities observed in OpenAI o3-mini high illustrate the benefits of advanced feedback integration layers, which can substantially boost performance after a prompt identifying weaknesses or inaccuracies. Large language models operating with numerous parameters may have the potential to outperform smaller systems, yet these findings highlight that scale alone does not guarantee superior clinical guidance. Cost and accessibility also merit attention: o3-mini high currently carries an operational cost roughly three-times that of DeepSeek-R1, which could restrict its adoption in smaller clinics or low-resource regions. Data curation, reward structures, and domain-specific fine-tuning appear to be equally important, if not more so, in determining real-world utility. Furthermore, some architectures, while excelling in raw capacity, may require additional refinement to manage domain-specific jargon, especially in a field as specialized as urology. The interplay between parameter efficiency and real-time adaptability remains intriguing—OpenAI o3-mini, though smaller, often displayed notable plasticity during iterative corrections, suggesting that carefully architected feedback loops can partially compensate for fewer parameters. The conclusion drawn from these results is that each system’s design priorities, such as maximizing interpretability or achieving a balance between speed and detail, shape its behavior in ways that are particularly salient in medical contexts. Continuous monitoring, versioning, and updates appear vital, given how guideline modifications can rapidly shift the landscape of best practices. Equally important is an understanding that domain knowledge, once accurately integrated, must be continuously tested against emerging evidence. From a systems-engineering standpoint, these models show that synergy among massive pre-training, strategic fine-tuning, and carefully structured reward signals can produce remarkable performance, but domain-based scrutiny is imperative to confirm reliability in patient-facing solutions.

A viewpoint from daily urology clinical practice emphasizes the pragmatic implications of these findings. Although thoroughness in addressing BPH symptomatology, diagnostic algorithms for UTIs, and cutting-edge management of prostate malignancies is important, the actual translation of model responses into better patient care necessitates contextual awareness and expert judgment. OpenAI o3-mini high, for instance, appears proficient at synthesizing guideline-based information on advanced oncologic treatments, such as novel hormonal therapies, offering clinicians rapid reinforcement of complex protocols. DeepSeek-R1’s robust baseline performance in patient counseling scenarios—particularly post-prostatectomy erectile dysfunction and fertility preservation—suggests a valuable utility for communicating nuanced information, as long as clinicians review the generated responses for accuracy. DeepSeek-V3 displays promise in certain logic-intensive queries, yet it occasionally fails to refine initial inaccuracies upon iterative prompting, which implies a risk of propagating misinformation if left unchecked. Meanwhile, OpenAI o3-mini demonstrates that an efficient, succinct style can be helpful when handling standard counseling points, yet it also demands supervision to ensure completeness in topics that require detailed elaboration. The scoring patterns observed in the study reflect not only raw content correctness but also an alignment with current recommendations from prominent urological associations. According to these findings, model selection may be guided by clinical priorities; for example, centers requiring extensive coverage of emerging oncologic guidelines might choose a higher-intensity reasoning model, whereas clinics focusing on rapid dissemination of conservative management tips might consider a concise generator. It remains prudent to maintain a skeptical stance when delegating educational tasks to generative systems, as even the strongest model can miss subtle changes in practice guidelines. Ensuring a human-in-the-loop approach is crucial for bridging knowledge gaps and mitigating potential harm. When it comes to patient counseling, a gentle but knowledgeable tone is required to handle sensitive topics such as psychosocial support in chronic prostatitis. Automated outputs should therefore be considered initial drafts, allowing the clinician to refine vocabulary, personalize care instructions, and address emotional factors that statistical algorithms cannot fully comprehend. Leveraging these models, if done responsibly, can improve workflow efficiency and aid in handling routine questions that otherwise consume precious consultation time. Yet, caution is advisable because oversight lapses may diminish the trust patients place in medical advice, especially if contradictions arise in subsequent consultations. The strength of this study is the integration of data comparing four well-known large language models, revealing clear differences in accuracy, self-correction, and adherence to guidelines. However, there are still limitations to this study. The sample size of this study is limited and inevitably relies on subjective expert scoring. Future research will require more multi-institutional studies and iterative improvements to ensure safe and effective application in urology practice.

## Conclusion

This comparative evaluation of DeepSeek-V3, DeepSeek-R1, OpenAI o3-mini, and OpenAI o3-mini high underscores both the promise and limitations of large language models in urological practice. By presenting 34 clinically oriented questions and an additional 25 guideline-based queries, and then employing blinded expert review, the study reveals that OpenAI o3-mini high achieves the highest overall scores in accuracy, detail, and alignment with recently updated guidelines. DeepSeek-R1 follows closely, particularly excelling in patient counseling domains, while DeepSeek-V3 demonstrates a solid baseline understanding but shows less flexibility in post-response self-corrections. OpenAI o3-mini, although generating more concise responses, proves surprisingly adept at iterative improvement when prompted to address inaccuracies, surpassing DeepSeek-V3 in this domain. These results indicate that a model’s capacity for comprehensive and guideline-driven responses is not solely dictated by response length or parameter count; rather, thoughtful design elements—such as feedback integration layers and domain-specific fine-tuning—can be decisive. Although such tools hold considerable promise for enhancing clinical workflows and patient education, expert oversight remains essential to correct nuanced errors and ensure alignment with rapidly evolving best practices. Future research should emphasize ongoing refinement, broader validation across diverse clinical settings, and cautious implementation to safeguard the standard of urological care.

## Electronic supplementary material

Below is the link to the electronic supplementary material.

Supplementary Material 1

Supplementary Material 2

## Data Availability

Data is provided within the manuscript or supplementary information files.
