# Supplementary material for: Comparative analysis of the performance of the large language models DeepSeek-V3, DeepSeek-R1, open AI-O3 mini and open AI-O3 mini high in urology
Source: World J Urol. 2025 Jul 7;43(1):416. doi: 10.1007/s00345-025-05757-4 (PMC12234633; doi:10.1007/s00345-025-05757-4)
Supplement: Supplementary file 1 — Supplementary Material 1 [file 345_2025_5757_MOESM1_ESM.docx]

**Appendix Table 2. List of 25 Guideline-Based Urology Questions Reflecting Recent Updates (Past 18 Months).**

| **Question ID** | **Topic / Guidelines** | **Sample Guideline-Focused Question** | **Recent Change or Update** |
| --- | --- | --- | --- |
| GB1 | Prostate Cancer Screening & Follow-up | What are the current age-specific PSA thresholds and follow-up intervals recommended by major urological associations for early detection of prostate cancer? | New risk stratification guidelines suggest individualized screening intervals, especially in men with higher genetic risks or strong family history. |
|  |  |  |  |
| GB2 | BPH Medication Updates | What do the latest guidelines advise regarding long-term use of combination therapy (α-blocker + 5α-reductase inhibitor) in moderate-severe LUTS? | Recent consensus highlights improved symptom control, but also addresses side effects such as sexual dysfunction and hypotension. |
| GB3 | Antibiotic Prophylaxis | Have there been changes to antibiotic prophylaxis duration for transurethral interventions in light of rising antimicrobial resistance patterns? | Updated guidance suggests shorter prophylaxis duration pre-/post-cystoscopy and more restrictive use to prevent resistance. |
| GB4 | Active Surveillance in Prostate Cancer | What modifications to Gleason score thresholds have been recommended for selecting men with low-risk prostate cancer for active surveillance? | Shifts emphasize MRI findings alongside Gleason ≤6 or 3+4 with minimal involvement, refining patient selection criteria. |
| GB5 | High-risk Prostate Cancer Management | According to recent guidance, how should androgen deprivation therapy (ADT) be sequenced with novel hormonal agents in high-risk localized or locally advanced prostate cancer? | Clarification on combination therapies (e.g., ADT + abiraterone) in high-risk disease before or after radiation. |
| GB6 | Metastatic Prostate Cancer Imaging | What advanced imaging modalities (PSMA PET/CT, choline PET/CT) are recommended for restaging metastatic prostate cancer according to the latest guidelines? | Guidelines increasingly advocate PSMA PET/CT for earlier detection of metastatic foci and therapy response. |
| GB7 | Neoadjuvant Therapy for Muscle‑Invasive Bladder Cancer (MIBC) | Do recent urological oncology guidelines support neoadjuvant immunotherapy in MIBC, and if so, which agents are recommended? | Emerging evidence supports checkpoint inhibitors combined with chemotherapy, though final approvals vary by region. |
| GB8 | UTIs in Special Populations | What new recommendations exist for antibiotic prophylaxis in patients with recurrent UTIs who are pregnant or who have indwelling catheters? | Shorter regimens and narrower-spectrum antibiotics are favored to reduce adverse events and resistance. |
| GB9 | Stone Disease Imaging | Have guidelines changed regarding imaging intervals and modality (low-dose CT vs. ultrasound) for surveillance of recurrent nephrolithiasis? | Recent guidelines suggest flexible intervals based on stone composition and recurrence risk; ultrasound is preferred in certain patients to reduce radiation exposure. |
| GB10 | Medical Expulsive Therapy in Urolithiasis | What updates exist on α-blocker therapy duration for ureteral stones, and under which conditions do guidelines suggest continuing beyond four weeks? | Guidelines increasingly emphasize a 2–4 week course, extended if the stone is large or passing slowly, but also caution about side effects. |
| GB11 | Imaging Follow-up After Prostate Surgery | How frequently should multiparametric MRI be used for surveillance of post‑prostatectomy patients with early biochemical recurrence according to new guidelines? | There is stronger endorsement for early MRI in men with rising PSA following radical prostatectomy to detect recurrence at low PSA levels. |
| GB12 | Hormonal Therapy Intervals | Have recommendations for extended vs. intermittent ADT changed in men with biochemical recurrence but without overt metastasis? | Some guidelines propose intermittent ADT in select patients to reduce side effects while maintaining efficacy. |
| GB13 | CRPC Management | What new data exist on combining radioligand therapy with novel anti-androgens for metastatic CRPC? | Potential synergy is highlighted, but patient selection and toxicity monitoring remain crucial. |
| GB14 | BPH and LUTS in Older Adults | Have there been updates regarding polypharmacy concerns for older adults with LUTS, especially around anticholinergics? | Guidelines caution about cognitive side effects and advocate alternative agents or dose adjustments in the elderly. |
| GB15 | Minimally Invasive Bladder Cancer Treatment | Are there new recommendations supporting novel intravesical therapies (e.g., gene therapy, newer strains of BCG) for high-risk non-muscle-invasive bladder cancer? | Investigational therapies are mentioned, but official endorsement remains limited to specific clinical trial settings. |
| GB16 | Stone Prevention in Metabolic Syndrome | What dietary and pharmacologic recommendations have been updated for stone prevention in patients with obesity or metabolic syndrome? | More stringent fluid goals and customized interventions (e.g., citrate supplementation), underscoring the role of weight reduction. |
| GB17 | Prostate Biopsy Techniques | Have guidelines changed regarding systematic vs. targeted MRI/ultrasound-fusion biopsy for initial evaluation of suspected prostate cancer? | Targeted MRI biopsies have become standard for suspected lesions, reducing unnecessary sampling. |
| GB18 | UTI Antimicrobial Stewardship | What new stewardship protocols are recommended for outpatient antibiotic selection in uncomplicated UTIs to minimize resistance? | Preference for narrow-spectrum agents (e.g., nitrofurantoin, fosfomycin) with short-course regimens where feasible. |
| GB19 | Postoperative Urological Care | Are there fresh recommendations for enhanced recovery after surgery (ERAS) protocols in major urologic surgeries (prostatectomy, cystectomy)? | Emphasizes multimodal analgesia, early mobilization, and judicious fluid management to shorten hospital stays. |
| GB20 | Overactive Bladder (OAB) and New Therapies | Are there new guidelines endorsing the use of novel β3‑adrenoceptor agonists or neuromodulation for refractory OAB? | Additional supportive evidence for β3‑agonists, but cost and patient selection remain considerations. |
| GB21 | Penile Rehabilitation After Prostate Surgery | Have guideline updates specified optimal timing and protocols for PDE5 inhibitor therapy post–radical prostatectomy? | Earlier initiation of PDE5 inhibitors and vacuum device therapy may enhance recovery, per some new recommendations. |
| GB22 | Advanced Imaging in Testicular Cancer | What changes have been recommended regarding the use of MRI or PET/CT in staging and follow-up of nonseminomatous germ cell tumors (NSGCT)? | Enhanced imaging protocols with PET/CT for certain residual masses; still debated for routine follow-up in stage I disease. |
| GB23 | Post-TURP Syndrome Prevention and Management | Have there been revisions to fluid selection or operative time recommendations to reduce risk of TURP syndrome? | Some guidelines now endorse iso-osmolar irrigants and strict time limits to reduce fluid absorption issues. |
| GB24 | Antibiotic Prophylaxis in Urodynamics | Do updated guidelines address prophylactic antibiotics for invasive urodynamics, especially in patients with recurrent UTIs or immunocompromise? | Yes, briefer prophylaxis is recommended only for high-risk patients or those with a history of severe infections. |
| GB25 | Surveillance Intervals After Nephrolithiasis Intervention | How have follow-up intervals and imaging recommendations changed post-URS or SWL in patients with complicated stone disease? | Shorter intervals for high-risk stone formers; routine imaging earlier to detect residual fragments or new stones. |
