# Supplementary material for: Comparative analysis of the performance of the large language models DeepSeek-V3, DeepSeek-R1, open AI-O3 mini and open AI-O3 mini high in urology
Source: World J Urol. 2025 Jul 7;43(1):416. doi: 10.1007/s00345-025-05757-4 (PMC12234633; doi:10.1007/s00345-025-05757-4)
Supplement: Supplementary file 2 — Supplementary Material 2 [file 345_2025_5757_MOESM2_ESM.docx]

**Appendix Table 1. List of 34 General Urology Questions Organized by Thematic Category.**

| **Question ID** | **Category** | **Sample Clinical Question** | **Clinical Relevance** |
| --- | --- | --- | --- |
| G1 | Pathology & Mechanisms | What hormonal and cellular mechanisms contribute to the development and progression of benign prostatic hyperplasia (BPH)? | Explores pathophysiologic underpinnings of BPH, informing targeted therapies such as 5α-reductase inhibitors and α-blockers. |
| G2 | Pathology & Mechanisms | How do urinary stone composition and metabolic factors interplay in nephrolithiasis formation? | Essential for tailoring preventive measures (e.g., dietary adjustments, medical expulsive therapy). |
| G3 | Pathology & Mechanisms | What role do chronic bacterial and inflammatory pathways play in recurrent urinary tract infections (UTIs)? | Helps identify underlying issues such as incomplete bladder emptying or bacterial biofilms. |
| G4 | Pathology & Mechanisms | What molecular mechanisms are implicated in the early stages of prostate cancer development? | Highlights genetic and epigenetic changes for risk stratification. |
| G5 | Pathology & Mechanisms | How do pelvic floor muscle dysfunctions lead to urinary incontinence post‑prostatectomy? | Emphasizes the anatomic and neuromuscular contributors to incontinence, aiding in postoperative rehabilitation. |
| G6 | Diagnosis | What are the indications and interpretation limits of serum PSA in routine prostate cancer screening? | Clarifies risk stratification, false positives, and age-specific cutoffs. |
| G7 | Diagnosis | How do modern imaging techniques (MRI fusion biopsy) improve prostate cancer detection and staging accuracy? | Stresses the shift toward targeted biopsies to reduce overdiagnosis. |
| G8 | Diagnosis | What diagnostic approach is recommended for adult male patients presenting with lower urinary tract symptoms (LUTS)? | Covers symptom scoring, physical exam, urinalysis, and imaging. |
| G9 | Diagnosis | Which clinical parameters help differentiate complicated from uncomplicated UTIs in outpatient settings? | Assists in deciding empiric antibiotic coverage and referral. |
| G10 | Diagnosis | What noninvasive methods are recommended to evaluate recurrent nephrolithiasis and stone burden? | Outlines the role of ultrasound, KUB radiograph, and low-dose CT. |
| G11 | Diagnosis | How should bladder outlet obstruction (BOO) be assessed in males presenting with obstructive voiding symptoms? | Covers uroflowmetry, PVR measurement, and possibly pressure-flow studies. |
| G12 | Treatment | What are the indications for transurethral resection of the prostate (TURP) vs. minimally invasive surgical therapies? | Guides intervention choice based on prostate size, comorbidities, and patient preference. |
| G13 | Treatment | How effective are α1-blockers compared to 5α-reductase inhibitors in controlling LUTS related to BPH? | Provides comparative data on efficacy, adverse events, and combination therapy. |
| G14 | Treatment | When should active surveillance be considered vs. immediate treatment for localized prostate cancer? | Discusses patient selection factors (Gleason score, PSA, MRI findings) for low-risk disease. |
| G15 | Treatment | What are the latest surgical and shock wave lithotripsy (SWL) options for large (>10 mm) kidney stones? | Reviews modality selection, success rates, and complications. |
| G16 | Treatment | How does combination therapy with PDE5 inhibitors and α-blockers benefit LUTS in men with erectile dysfunction? | Addresses dual-treatment rationale and side-effect profiles. |
| G17 | Treatment | What systemic therapies are recommended for castration-resistant prostate cancer (CRPC)? | Summarizes the role of novel anti-androgens, chemotherapy, and radioligand therapy. |
| G18 | Treatment | How should antibiotic regimens be escalated in cases of persistent UTIs unresponsive to first-line agents? | Highlights resistance patterns, culture-directed therapy, and stewardship principles. |
| G19 | Treatment | What pain management strategies are recommended for acute renal colic caused by nephrolithiasis? | Reviews NSAIDs, opioids, α-blockers, and hydration protocols. |
| G20 | Prevention | What dietary modifications reduce the risk of recurrent nephrolithiasis? | Discusses fluid intake, oxalate restriction, and calcium moderation. |
| G21 | Prevention | How does prophylactic antibiotic use differ between simple cystoscopy and more invasive urological procedures? | Clarifies guidelines for antibiotic stewardship in urologic interventions. |
| G22 | Prevention | Which preventive measures help reduce rates of catheter‑associated urinary tract infections (CAUTIs)? | Emphasizes sterile technique, minimizing catheter duration, and using coated catheters. |
| G23 | Prevention | How does lifestyle intervention (exercise, weight loss, smoking cessation) impact BPH progression? | Discusses modifiable risk factors and overall urologic health benefits. |
| G24 | Prevention | How do current immunization guidelines impact prophylaxis against urosepsis in high-risk patients? | Covers recent vaccine developments (e.g., influenza, pneumococcal) that may lower complications. |
| G25 | Prevention | What are the recommended follow-up intervals for surveillance imaging post-kidney stone passage? | Focuses on preventing future episodes and evaluating stone recurrence risk. |
| G26 | Patient Counseling | How should patients be counseled regarding postoperative erectile dysfunction after radical prostatectomy? | Outlines realistic expectations, rehabilitation protocols, and partner involvement. |
| G27 | Patient Counseling | What are the main points to address when educating men on fertility preservation prior to testicular cancer therapy? | Discusses sperm banking, fertility-sparing options, and potential gonadotoxic treatments. |
| G28 | Patient Counseling | How can lifestyle modifications and medication adherence be reinforced in BPH management? | Encourages patient education on fluid management, medication schedules, and follow-up compliance. |
| G29 | Patient Counseling | What is the recommended counseling approach for male patients with recurrent UTIs regarding sexual practices and hygiene? | Discusses prophylactic measures, hydration, and potential benefit of cranberry derivatives. |
| G30 | Patient Counseling | Which communication strategies help patients understand the pros/cons of PSA screening? | Emphasizes shared decision-making, highlighting benefits vs. risks of overdiagnosis and overtreatment. |
| G31 | Patient Counseling | How should lifestyle changes be approached in counseling men with early-stage prostate cancer opting for active surveillance? | Reinforces importance of exercise, healthy diet, smoking cessation, and follow-up testing. |
| G32 | Patient Counseling | What key information should be provided to patients with CRPC regarding treatment side effects and prognosis? | Informs on fatigue, bone health, psychological support, and palliative care options. |
| G33 | Patient Counseling | How should psychosocial support for patients with chronic prostatitis/chronic pelvic pain syndrome be integrated? | Addresses mental health, stress management, and quality-of-life concerns. |
| G34 | Patient Counseling | Which factors must be highlighted for patients with recurrent stones regarding fluid intake and medical therapy compliance? | Reinforces fluid goals, medication side effects, and importance of regular follow-up. |
